# Preparation of Agricultural Jiaosu from vegetable waste: multi-dimensional effects of microbial inoculants on the properties of Agricultural Jiaosu and fertilizer efficiency

**DOI:** 10.3389/fmicb.2025.1576663

**Published:** 2025-06-18

**Authors:** Zhuo Chen, Xiaocui Liu, Tiantian Ban, Chao Ma

**Affiliations:** Guizhou Province Horticulture Engineering Technology Research Center, Institute of Horticulture, Guizhou Academy of Agricultural Sciences, Guiyang, China

**Keywords:** Agricultural Jiaosu, Chinese cabbage, microbial diversity, organic acids, nutrient elements

## Abstract

**Purpose of the study:** To explore the effects of adding different bacterial agents before fermentation on the Agricultural Jiaosu derived from waste from Chinese cabbage.

**Material and methods:** This study used the waste from Chinese cabbage as the raw material and added *Lactobacillus*, *Acetobacter*, *Yeast*, and *Bacillus subtilis* respectively for fermentation treatment. Systematic research was conducted on the effects of different bacterial agents on the nutritional elements, microbial diversity, organic acid content of Agricultural Jiaosu, as well as their impacts on the growth and yield of Chinese cabbage.

**Results:** The results showed that there were significant differences in the above aspects among the treatments with different bacterial agents. In terms of nutritional elements, the treatment group with *Lactobacillus* addition had the highest total carbon, total nitrogen, and total phosphorus contents; the treatment group with *Acetobacter* addition had the highest total potassium and carbon-nitrogen ratio. Microbial diversity analysis revealed that the relative abundance of *Bacteroides* was higher in the *Lactobacillus* treatment group; *Arcobacter* and *Vishniacozyma* were relatively more abundant in the *Acetobacter* and *Bacillus subtilis* treatment groups; and the relative abundance of *Lysinibacillus* was higher in the *Yeast* treatment group. Organic acid content analysis indicated that the *benzoic acid* content was higher in the *Lactobacillus* treatment group; the *benzenepropanoic acid*, *azelaic acid*, and *pyroglutamic acid* contents were higher in the *Acetobacter* treatment group; the *succinic acid* content was the highest in the *Yeast* treatment group; and the *glutaric acid* content was the highest in the *Bacillus subtilis* treatment group. Moreover, the effects of the *Yeast* and *Acetobacter* treatment groups on promoting the growth of Chinese cabbage were more significant.

**Conclusion and future prospective:** In conclusion, Agricultural Jiaosu is rich in abundant nutritional elements, diverse microbial communities, and various organic acids, and has a significant fertilizer effect on Chinese cabbage production. Adding different bacterial agents before fermentation leads to differences in the composition and function of the finished Agricultural Jiaosu products. Therefore, appropriate bacterial agents can be selected for fermentation according to specific application goals to optimize the performance and application effect of Agricultural Jiaosu.

## 1 Introduction

Over the past 4 decades, China’s vegetable production has increased significantly with the increase in productivity(Tang et al., 2023). According to the data of the National Bureau of Statistics of China, the annual output of vegetables in China has reached 828.6811 million tons in 2023 (National Bureau of Statistics of China, 2023). If the loss in the process of vegetable harvesting and transportation is calculated according to the loss rate of 30%, about 24,860.433 million tons of vegetable waste will be produced in a year. If such a large amount of vegetable waste is not properly disposed, it will lead to environmental problems such as pollution of water and soil, production of odor, and breeding of pathogenic bacteria (Lu et al., 2022; Wan et al., 2020; Wang et al., 2019). Therefore, how to effectively dispose vegetable waste is a crucial issue in the sustainable development of agriculture.

It is also found in the data of the National Bureau of Statistics of China that the net amount of agricultural chemical fertilizer applied in 2023 reaches 50.217,400 tons (National Bureau of Statistics of China, 2023). Long-term excessive application of chemical fertilizers can lead to water eutrophication, increase greenhouse gas emissions, decrease biodiversity, damage soil structure, reduce the number of beneficial microorganisms in soil, lead to soil acidification and salinization, and ultimately reduce soil fertility and crop yield. These environmental problems not only affect agricultural productivity, but also have long-term impacts on human health and ecosystems (Grizzetti et al., 2022; Zhang et al., 2021). Conversion of vegetable waste into biofertilizers offers a sustainable strategy to divert organic waste from landfills while reducing reliance on synthetic fertilizers, achieving dual environmental and agronomic benefits.

The fertilizer utilization of vegetable waste is mainly divided into aerobic composting and anaerobic fermentation (Zaini et al., 2023). However, organic carbon in aerobic composting is mainly emitted in the form of carbon dioxide, methane and nitrous oxide. If all vegetable wastes are treated by aerobic composting, it will cause a large amount of carbon emissions, which is the main cause of global warming, ecological imbalance, technical, economic and social problems (Ye et al., 2023; Abeydeera et al., 2019). If anaerobic fermentation is adopted, it can effectively reduce greenhouse gas emissions and promote carbon neutrality compared with aerobic composting because it should be sealed during fermentation (Duan et al., 2025).

In recent couple of years several researcher have been working on anaerobic fermentation of agricultural waste. Previous studies have shown that biofertilizers made from agricultural wastes can effectively promote plant growth, improve nutrient utilization rate, and enhance plant stress resistance. Moreover, anaerobic fermentation of fruit and vegetable wastes with specific raw materials can also effectively improve the yield of volatile fatty acids (Puglia et al., 2021; Xing et al., 2020). Research on Agricultural Jiaosu by China Agricultural University has shown that Agricultural Jiaosu produced by anaerobic fermentation of agricultural waste cannot only be used as fertilizer, but also be used as bacteriostatic agent to prevent pests and diseases in agricultural production (Gao et al., 2022; Zhang et al., 2020). Gao et al.’s (2023) study showed that the fermentation of Agricultural Jiaosu is a process that converts organic matter into organic acids and other metabolites, mainly including hydrolysis, acid production and ripening stages, In this process, microorganisms play a major role in fermentation, and the dominant bacteria, organic acids and other metabolites in Agricultural Jiaosu fermented on different substrates are different. Based on this, we began to think about the diversity of Agricultural Jiaosu fermentation. The microbial diversity, organic acids and nutrients of Agricultural Jiaosu fermented on different substrates are different. If the same substrate is used for fermentation, a small amount of different bacterial agents are added artificially. Is it possible to produce different Agricultural Jiaosu? What kind of influence will it have on microbial diversity, organic acids, nutrients and fertilizer efficiency in the field of Agricultural Jiaosu? After understanding this point, it is of great significance for the preparation and precise application of Agricultural Jiaosu with the same substrate.

Therefore, in this study, four different bacterial agents were selected and added to Agricultural Jiaosu fermented with Chinese cabbage as the substrate before fermentation. The four bacterial agents selected were lactic acid bacteria, acetic acid bacteria, *Yeast*s and *Bacillus subtilis*. Lactic acid bacteria (*Lactobacillus* spp.) and acetic acid bacteria (*Acetobacter* spp.) were selected as inoculants based on their reported dominance in leafy vegetable waste fermentation by Gao et al. (2023), where these taxa exhibited robust carbohydrate metabolism and organic acid production., which are critical for enhancing the antimicrobial activity and nutrient retention of Agricultural Jiaosu. *Yeast* was selected because *Yeast* is often used in food fermentation, such as wine making or baking. In this study, we tried to use it in the fermentation of Agricultural Jiaosu to observe its effect. *Bacillus subtilis* is widely used in agriculture, industry, biomaterials, and medicine. Adding appropriate amount of *Bacillus subtilis* to compost can significantly increase the humus and carbon content of compost, thereby improving soil quality and promoting crop growth (Su et al., 2020). Therefore, this study wanted to try to use it in the fermentation of Agricultural Jiaosu to see what kind of reaction would occur. In this study, different bacterial agents were added before Agricultural Jiaosu fermentation to study how a small amount of exogenous addition of different bacterial agents would affect the finished product of Agricultural Jiaosu, and to determine the best bacterial agent. By linking microbial consortia composition to Agricultural Jiaosu functionality, this work delivers a biofertilizer optimization protocol that bridges organic waste recycling and agroecological intensification.

## 2 Materials and methods

### 2.1 Agricultural Jiaosu sample preparation

With Chinese cabbage waste as raw material, the raw material was weighed according to the ratio of brown sugar, Chinese cabbage waste and water of 1:*3*:10. The specific operation is to take 6 kg of Chinese cabbage waste and mince it, put it into a plastic bucket, add 2 kg of brown sugar, then inject 20 L of water, and finally add 10 g of different bacterial agents (lactic acid bacteria, acetic acid bacteria, *Yeast*, and *Bacillus subtilis*) into different plastic buckets, and stir it well. Reserve 30% of the fermentation space in the plastic bucket, tighten the lid, and place it in a cool and ventilated place in the room until fermentation. The fermentation time is about 90 days, then Agricultural Jiaosu can be obtained (Zhang et al., 2020). Except for different bacterial agents, the other steps and fermentation conditions were the same, and each treatment was repeated three times.

The samples with different microbial agents were numbered as follows: A- lactic acid bacteria; B- acetic acid bacteria; C- *Yeast*; D- *Bacillus subtilis*.

### 2.2 Determination of total carbon, total nitrogen, total phosphorus, and total *potassium* contents in Agricultural Jiaosu

Total nitrogen was detected by TOC analyzer (ASTM International, 2016), the sample was subjected to catalytic thermal decomposition at high temperature, nitrogen was oxidized to nitric oxide, and the generated nitric oxide was detected by chemiluminescence detector and converted into optical signal, and then the total nitrogen content was measured. The total carbon was detected by TOC analyzer (Shetty and Goyal, 2022), and the sample reacted with oxygen at high temperature to release CO_2_. The total carbon content in the unknown sample was obtained by measuring the content of CO_2_. Total phosphorus was detected by *potassium* persulfate oxidation molybdenum blue spectrophotometry (Cook and Daughton, 1981). *Potassium* sulfate was added to the water sample, and organic phosphorus in the water was converted to orthophosphorus under high temperature and high pressure in a closed container. Orthophosphorus showed blue color with ammonium molybdate phosphomolysomolybdate heteropolysate under certain acidity and reducing agent conditions. Its absorbance value is proportional to its concentration. Phosphorus content could be determined by colorimetry. Total *potassium* was detected by flame atomic absorption spectrophotometry (Chekri et al., 2010), and the basic principle is to measure the absorption of resonant radiation by ground-state atoms. In a high temperature flame, *potassium* and sodium are easily ionized, thus reducing the number of ground state atoms involved in atomic absorption. In particular, *potassium* is more obvious when the concentration is low. Generally, sodium is higher than *potassium* in water, and a large amount of sodium has a sensitizing effect on *potassium*. To overcome this phenomenon, cesium, which is more easily ionized than *potassium*, is added as an ionization buffer to provide enough electrons to shift the ionization balance toward the formation of ground-state atoms, at which point *potassium* and sodium can be measured continuously in the same sample.

### 2.3 Bacterial diversity analysis

16S rRNA is located on the small ribosomal subunit of prokaryotic cells, including 10 Conserved Regions and 9 Hypervariable Regions. The conserved regions are not significantly different among bacteria, and the hypervariable regions are genus or species specific. There are some differences depending on the kinship. Therefore, 16S rDNA can be used as a characteristic nucleic acid sequence to reveal biological species and is considered to be the most suitable indicator for bacterial phylogenetic and taxonomic identification (Pace, 1997). 16S rRNA Amplicon Sequencing usually selects one or several variant regions, uses the conserved region to design universal primers for PCR amplification, and then conducts sequencing analysis and strain identification of the hypervariable region. 16S rRNA amplicon sequencing technology has become an important tool to study the composition and structure of microbial communities in environmental samples. According to the characteristics of the amplified 16S region, a small fragment library was constructed and Paired end sequencing was performed on the Illumina NovaSeq sequencing platform. Representative sequences were generated by Reads splicing and filtering, clustering or noise reduction methods, which could be used for species annotation and abundance analysis. Through Alpha Diversity and Beta Diversity analysis, the differences in species composition and community structure among samples can be revealed, and personalized analysis and deep data mining can be conducted (Caporaso et al., 2011; Youssef et al., 2009; Hess et al., 2011). Specific sequencing methods were as follows: CTAB or SDS methods were used to extract genomic DNA from the samples, and then agarose gel electrophoresis was used to detect the purity and concentration of DNA (Doyle and Doyle, 1987). An appropriate amount of sample DNA was taken in a centrifuge tube, and the sample was diluted to 1 ng/μL with sterile water. Using diluted genomic DNA as a template, specific primers with Barcode were used depending on the region selected for sequencing, Phusion^®^ High-Fidelity PCR Master Mix with GC Buffer from New England Biolabs, and high-performance high-fidelity enzyme were used for PCR to ensure amplification efficiency and accuracy. Primers corresponding to the region: 16S V4 primers (515F and 806R): identify bacterial diversity; Then the pooled and purified PCR products were analyzed by electrophoresis on 2% agarose gel. The qualified PCR products were purified by magnetic beads and quantified by enzyme labeling. The samples were mixed according to the concentration of PCR products, and the PCR products were detected by 2% agarose gel electrophoresis after fully mixing. The TruSeq^®^ DNA PCR-Free Sample Preparation Kit was used for library construction. The constructed library was quantified by Qubit and Q-PCR, and the library was qualified. On-machine sequencing was performed using NovaSeq6000.

### 2.4 Analysis of fungal diversity

Fungi are usually invisible to the naked eye, but they play an important role in most life processes above and below the surface, in plants and animals. The traditional classification and identification of fungi are mainly based on their morphological, growth, physiological and biochemical characteristics. There are many kinds of fungi. According to the estimates of scientists, the total number of fungal species on the Earth is between 2.2 million and 3.8 million, but the individual polymorphism of fungi is not obvious, and the physiological and biochemical characteristics are unstable with the change of the environment. Therefore, there are great limitations in using traditional methods to classify fungi. With the development of molecular biology technology, nucleic acid sequence analysis has been widely used in fungal classification and identification. The commonly used technique is ITS (Internal Transcribed Spacer) sequencing (Oros-Sichler and Smalla, 2013; Bengtsson-Palme et al., 2013; Findley et al., 2013). Two fungi, ITS1 and ITS2, are commonly used for taxonomic studies. ITS1 is located between 18S and 5.8S of the eukaryotic ribosomal rDNA sequence, and ITS2 is located between 5.8S and 28S. Since it does not need to add mature ribosomes, it can withstand more variation during evolution. The evolution rate of this rDNA is 10 times that of 18S rDNA, and it belongs to the moderately conserved region. Paired end sequencing of ITS1 or ITS2 regions using the Illumina NovaSeq platform has the characteristics of high sequencing depth and conducive to the identification of species in low-abundance communities. It is the best choice for the study of fungal community diversity (Degnan and Ochman, 2012; Caporaso et al., 2012). CTAB or SDS methods were used to extract genomic DNA from the samples, and then agarose gel electrophoresis was used to detect the purity and concentration of DNA. An appropriate amount of sample DNA was taken in a centrifuge tube, and the sample was diluted to 1 ng/μL with sterile water. Using diluted genomic DNA as a template, specific primers with Barcode were used depending on the region selected for sequencing, Phusion^®^ High-Fidelity PCR Master Mix with GC Buffer from NewEngland Biolabs, and high-efficiency high-fidelity enzyme were used for PCR to ensure amplification efficiency and accuracy. The primers of ITS1 (ITS5-1737F and ITS2-2043R) were used to identify fungal diversity. The PCR products were then pooled and purified, and the PCR products were detected by electrophoresis on 2% agarose gels. The qualified PCR products were purified by magnetic beads and quantified by enzyme labeling. The samples were mixed according to the concentration of PCR products, and the PCR products were detected by 2% agarose gel electrophoresis after fully mixing. The TruSeq^®^ DNA PCR-Free Sample Preparation Kit was used for library construction. The constructed library was quantified by Qubit and Q-PCR, and the library was qualified. On-machine sequencing was performed using NovaSeq6000.

### 2.5 Organic acid analysis

Organic acids refer to a class of organic compounds with acidity. The most common organic acids are carboxylic acids. In addition, sulfinic acid, sulfonic acid, thiocarboxylic acid, etc. also belong to organic acids. In animals and plants, except for a few organic acids that exist in a free state, they generally exist in the form of salts or esters by plasma combination with *potassium*, sodium, and calcium. Many organic acids can directly participate in biochemical reactions in the process of life activities and play a very important role. This study uses the LC-MS/MS platform based organic acid testing and quantitative analysis method, the specific operation steps are as follows.

#### 2.5.1 Chemicals and reagents

Methanol (MeOH) was purchased from Merck (Darmstadt, Germany). MilliQ water (Millipore, Bradford, United Kingdom) was used in all experiments. All of the standards were purchased from Sigma-Aldrich (St. Louis, MO, United States). Formic acid was bought from Sigma-Aldrich (St. Louis, MO, United States). The stock solutions of standards were prepared at the concentration of 1 mg/mL in MeOH. All stock solutions were stored at −*20*°C. The stock solutions were diluted with MeOH to working solutions before analysis.

#### 2.5.2 Sample preparation and extraction

Take 2 mL of the sample, freeze-dry it, add 1,000 μL of 70% methanol aqueous to re-dissolve, and vortex it for 5 min. Centrifuge at 12,000 r/min for 10 min at 4°C, 250 μL of the supernatant was collected. The supernatant was again centrifuged at 12,000 r/min for 5 min at 4°C, 180 μL of the supernatant was transferred for further LC-MS analysis.

#### 2.5.3 UPLC conditions

The sample extracts were analyzed using an LC-ESI-MS/MS system (UPLC, ExionLC AD, MS,^1^ QTRAP^®^ 6500 + System).^2^ The analytical conditions were as follows, HPLC: column, ACQUITY HSS T3 (i.d.2.1 × 100 mm, 1.8 μm); solvent system, water 0.05% formic acid (A), acetonitrile with 0.05% formic acid (B); The gradient was started at 5% B (0 min), increased to 95% B (8–9.5 min), finaly ramped back to 5% B (9.6–12 min); flow rate, 0.35 mL/min; temperature, 40°C; injection volume: 2μL.

#### 2.5.4 ESI-MS/MS

AB 6500 + QTRAP^®^ LC-MS/MS System, equipped with an ESI Turbo Ion-Spray interface, operating in both positive and negative ion modes and controlled by Analyst 1.6 software (AB Sciex). The ESI source operation parameters were as follows: ion source, turbo spray; source temperature 550°C; ionspray voltage (IS) 5,500 V (Positive), −4,500 V (Negative); curtain gas (CUR) were set at 35.0 psi; DP and CE for individual MRM transitions was done with further DP and CE optimization. A specific set of MRM transitions were monitored for each period according to the organic acid eluted within this period.

### 2.6 Effects of Agricultural Jiaosu supplemented with different microbial agents on growth and yield of Chinese cabbage

#### 2.6.1 The vegetable variety

“Hanqing Chi Chinese Cabbage” is a first-generation hybrid of chibok cabbage cultivated by the male sterile line. The plant type is orderly and uniform, upright and compact, strong winter, wrinkled leaf surface, strong growth, green side leaves tender green, and a single ball weighs about 2 kg. Soft leaf rate 59%, clean vegetable rate 87%, spherical yellow, sweet flavor.

#### 2.6.2 Agricultural Jiaosu and fertilizer

Test fertilizer: compound fertilizer (N-P-K content 15%-15%-15%).

Agricultural Jiaosu: Agricultural Jiaosu is produced by fermentation of Chinese cabbage waste, and there are four different types of Agricultural Jiaosu; the detailed preparation methods and types are shown in 2.1.

#### 2.6.3 Experimental treatment

There were six experimental treatments, including A-lactic acid bacteria; B-acetic acid bacteria; C- *Yeast*; D- *Bacillus subtilis*; E-Compound Fertilizer; CK- No fertilization.

#### 2.6.4 Experimental site

Experimental Field of Guizhou Institute of Horticulture, Huaxi District, Guiyang City, Guizhou Province.

#### 2.6.5 Planting and fertilization conditions

In the way of seedling cultivation, the seeds were directly seeded into the 72-hole hole dish. The cultivation substrate used in the experiment was a uniform mixture of 20% perlite, 50% coconut shell and 30% peat. Sowing on September 27, 2024, seedling management was consistent, large cabbage seedlings were transplanted when they were 4–5 leaves, and colonized on November 5, 2024. Dried cattle manure (50 kg⋅m-3) was used as base fertilizer, and Fertilization or Agricultural Jiaosu was applied once on days 0, 20, and 40 after colonization.

#### 2.6.6 Measurement of indicators

Plant height, leaf length, leaf width, stem diameter and leaf number were measured directly, and each treatment was repeated three times. Plant height was measured from the stem base of Chinese cabbage to the top of the leaf column, leaf length was measured from the leaf tip of Chinese cabbage to the base of the leaf stalk, leaf width was measured from the width of the widest part of Chinese cabbage leaves, and stem diameter was measured from the stem of the plant. The number of leaves was counted by counting the total number of leaves with leaves larger than 3 cm. Fresh weight of the whole plant was measured directly at harvest. Plant height, leaf length, leaf width, stem diameter, leaf number and fresh weight were measured during the harvest period of Chinese cabbage.

## 3 Results

### 3.1 Analysis of the appearance, total nutrients, and C/N ratio of Agricultural Jiaosu

#### 3.1.1 Appearance analysis of Agricultural Jiaosu

**FIGURE 1:**
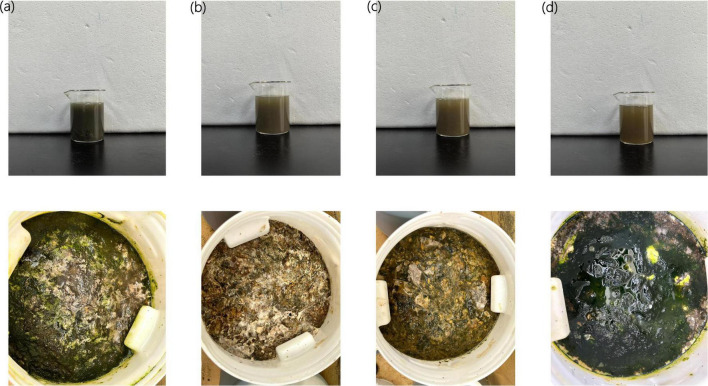
Agricultural Jiaosu fermented with four different bacterial agents was added before fermentation. lactic acid bacteria **(a)**; acetic acid bacteria **(b)**; *Yeast* **(c)**; *Bacillus subtilis* **(d)**; The top picture is a well-fermented Agricultural Jiaosu, and the bottom picture is a fermented Agricultural Jiaosu.

As shown in Figure 1, during the fermentation process of Agricultural Jiaosu with Chinese cabbage as raw material, the color of Agricultural Jiaosu with different bacterial agents was different. The color of Agricultural Jiaosu with lactic acid bacteria and *Bacillus subtilis* was darker. More like deep olive green; However, the color of Agricultural Jiaosu with acetic acid bacteria and with *Yeast* was lighter and more yellowish green. However, there was no significant difference in the color of the filtered Agricultural Jiaosu liquid after the fermentation was completed, which was deep olive green.

#### 3.1.2 Analysis of total nutrients and C/N ratios in Agricultural Jiaosu

As shown in Table 1, the total nutrient content of Agricultural Jiaosu fermented by adding different bacterial agents before fermentation was significantly different. With respect to the total carbon content of Agricultural Jiaosu, there were significant differences in each treatment, with the order of total carbon content from high to low being lactic acid bacteria treatment, acetic acid bacteria treatment, *Yeast* treatment, and *Bacillus* treatment. As for the total nitrogen content of Agricultural Jiaosu, the lactic acid bacteria treatment was significantly higher than that of acetic acid bacteria and *Yeast* treatments, which were significantly higher than that of *Bacillus* treatment. As for the total phosphorus content of Agricultural Jiaosu, the lactic acid bacteria treatment was significantly higher than that of acetic acid bacteria and *Yeast* treatments, which were significantly higher than that of *Bacillus* treatment. As for the total *potassium* content of Agricultural Jiaosu, the acetic acid bacteria treatment was significantly higher than the *Yeast* and lactic acid bacteria treatments, and the *Yeast* and lactic acid bacteria treatments were significantly higher than the *Bacillus* treatment. Agricultural Jiaosu with appropriate C/N ratio could promote microbial growth and reproduction, affect soil fertility, and promote plant growth. However, the C/N of this batch of Agricultural Jiaosu fermented with Chinese cabbage as raw material was between 4 and 5, and the C/N of lactic acid bacteria and acetic acid bacteria treatments was significantly higher than that of *Yeast* and *Bacillus* treatments.

**TABLE 1:** Total nutrient content and C/N ratio of Agricultural Jiaosu in different treatments.

Sample	Total carbon (mg/L)	Total nitrogen (mg/L)	Total Phosphorus (mg/L)	Total kaliu (mg/L)	Carbon nitrogen ratio (mg/L)
A	2689.6 ± 4.996a	576.533 ± 1.69a	87.683 ± 0.895a	530.56 ± 1.206b	4.667 ± 0.006a
B	2233.6 ± 13.363b	483.973 ± 11.503b	72.517 ± 1.354b	547.76 ± 1.526a	4.617 ± 0.101a
C	2108.8 ± 7.2c	480.24 ± 4.673b	71.47 ± 1.455b	534.1 ± 3.09b	4.39 ± 0.053b
D	1689.6 ± 2.884d	385.7333 ± 9.04c	53.777 ± 0.949c	508.14 ± 3.744c	4.38 ± 0.105b

If the data in the same column are significantly different at the 0.05 level, they will be expressed in different lowercase letters, the same as below.

### 3.2 Analysis of bacterial diversity in Agricultural Jiaosu

#### 3.2.1 Sequencing valid data and alpha diversity index

Table 2 indicates that for the Agricultural Jiaosu samples, the Effective Tags range from 60,654 to 111,682 and the Effective Bases range from 24,658,653 to 43,251,307. The Q20 and Q30 values are consistently above 90%, with GC content between 49.38 and 50.59%. These metrics suggest high sequencing accuracy and quality.

**TABLE 2:** Data preprocessing statistics and quality control analysis.

Sample name	Raw tags	Clean tags	Effective tags	Effective ratio (%)	Effective bases (nt)	Mean length	Q20 (%)	Q30 (%)	GC (%)
A1	93,385	92,559	82,968	88.85	34,787,538	419	97.07	91.73	50.49
A2	111,918	111,109	103,187	92.2	43,251,307	419	96.85	91.19	50.49
A3	105,014	104,187	92,455	88.04	38,697,841	419	97.07	91.8	50.7
B1	97,414	96,626	85,611	87.88	35,791,702	418	97.13	91.87	50.6
B2	61,095	60,654	58,836	96.3	24,658,653	419	97.24	92.14	50.59
B3	106,776	106,035	98,605	92.35	41,279,152	419	97.27	92.2	50.51
C1	106,577	105,802	95,740	89.83	40,165,511	420	96.97	91.42	50.54
C2	104,836	104,069	71,227	67.94	30,333,830	426	97.9	93.75	51.55
C3	103,616	102,950	95,629	92.29	40,048,972	419	97.18	91.91	51.04
D1	103,589	102,983	84,801	81.86	35,158,677	415	97.02	91.33	49.95
D2	112,382	111,682	99,736	88.75	41,355,816	415	97.13	91.8	49.9
D3	102,445	101,821	95,292	93.02	39,601,182	416	97.09	91.66	49.38

Table 3 reveals that the observed ASVs in these samples range from 230 to 350, with Shannon indices between 2.058 and 4.068 and Simpson indices ranging from 0.664 to 0.963. These results indicate a rich bacterial community with high species diversity and evenness. Additionally, the Good’s coverage index exceeds 0.999, confirming sufficient sequencing depth to adequately represent the entire bacterial community.

**TABLE 3:** Alpha diversity statistics table based on ASV.

Sample	Observed ASV	Shannon	Simpson	Chao1	ACE	Goods coverage
A1	350	3.957	0.961	358.5	355.967	1
A2	346	3.987	0.961	351.455	351.871	1
A3	348	4.068	0.963	351.64	353.38	1
B1	320	3.582	0.916	320.625	321.14	1
B2	308	3.54	0.922	308.429	308.872	1
B3	323	3.438	0.912	333	334.451	0.999
C1	320	3.808	0.944	329.731	331.039	0.999
C2	230	2.058	0.664	237.037	238.425	0.999
C3	288	3.693	0.931	296.55	297.204	0.999
D1	291	3.125	0.82	303.214	298.474	0.999
D2	289	2.836	0.766	292.391	293.398	1
D3	297	2.891	0.795	311.526	307.14	0.999

#### 3.2.2 Composition and differences in bacterial communities

**FIGURE 2:**
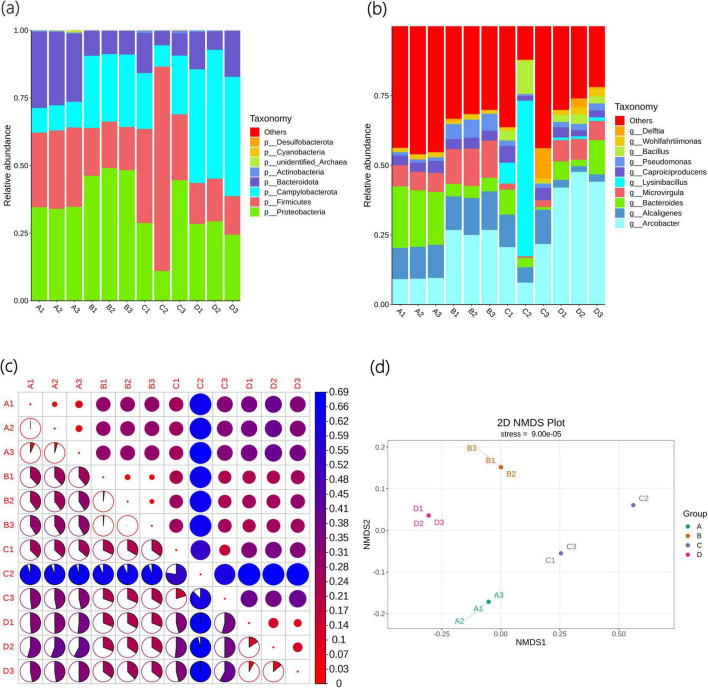
Beta diversity indices of bacterial communities of Agricultural Jiaosu samples after adding different bacterial agents at the phylum level **(a)**, at the genus level **(b)**, and based on ASV **(c)** heatmaps of Beta diversity indices, and **(d)** non-metric multi-dimensional scaling analysis.

As shown in Figure 2a, the top five bacterial phyla with the largest number of Agricultural Jiaosu sample types are *Pseudomonadota*, *Firmicutes*, *Campylobacterota*, *Bacteroidota*, and *Actinobacteria*. Among them, the abundance of *Pseudomonadota* in Group A and Group B is the highest, *Firmicutes* in Group C has the highest abundance, and *Campylobacterota* in Group D has the highest abundance. This indicates that they are the dominant phyla in Agricultural Jiaosu, and the dominant phyla will change depending on the different bacterial agents added before fermentation.

As shown in Figure 2b, the top 10 bacterial genera with the highest abundance are *Arcobacter*, Alcaligenes, *Bacteroides*, Microvirgula, *Lysinibacillus*, *Caproiciproducens*, *Caproiciproducens*, *Bacillus*, *Wohlfahrtiimonas*, and *Delftia*. The abundance of *Arcobacter*, Alcaligenes, *Bacteroides*, Microvirgula, and *Lysinibacillus* is the highest, indicating that they may be the dominant genera in Agricultural Jiaosu. The dominant genera in Group A are *Bacteroides*, in Group B are *Arcobacter*, in Group C are *Lysinibacillus*, and in Group D are *Arcobacter*. This indicates that the overall bacterial species in the Agricultural Jiaosu fermented from Chinese cabbage are similar, but the dominant genera will change after adding different bacterial agents before fermentation. All these dominant genera are helpful for agricultural production, but the specific mechanisms are different. *Bacteroides* mainly enhances soil fertility by decomposing complex organic matter and participating in nitrogen cycling in soil, while in plants, it regulates the rhizosphere environment and microbial community structure to promote plant growth and enhance stress resistance. *Arcobacter* has a relatively indirect role in soil and plants, mainly by decomposing organic matter and regulating nitrogen balance to provide a healthy soil environment for plants. *Lysinibacillus* has strong biocontrol ability and promotes plant growth in soil, and can directly promote plant growth and enhance plant stress resistance through multiple mechanisms. Therefore, the effects of different groups of Agricultural Jiaosu may also vary.

The Beta diversity index heatmap based on ASV is shown in Figure 2c. The circles in the upper triangular grid of the figure represent the beta diversity among samples. The smaller the circle, the redder the color, indicating that the beta diversity value is smaller and the diversity difference among samples is smaller; the colors and sizes of the circles in the lower triangular grid are consistent with those in the upper triangular grid in terms of meaning. There are significant differences in bacterial composition among the groups, but compared with other groups, the differences between Group B and Group D are relatively small, indicating that the bacterial composition of Group B and Group D is the most similar among the groups. In addition, the C2 sample may have experienced an unknown accidental event during the fermentation process, and the differences with other samples are very significant. These results are also similar to the Non-Metric Multi-Dimensional Scaling (NMDS) analysis shown in Figure 2d. NMDS is an ecological research method that can better reflect the non-linear structure of ecological data, and the distance between points can reflect the differences between samples. Each point in the figure represents a sample, and the distance between points represents the degree of difference, and samples in the same group are represented by the same color. When the Stress is less than 0.2, it indicates that NMDS can accurately reflect the degree of differences among samples, and the results are reliable.

### 3.3 Analysis of fungal diversity in Agricultural Jiaosu

#### 3.3.1 Sequencing valid data and alpha diversity index

Table 4 indicates that for the Agricultural Jiaosu samples, the Effective Tags range from 62,018 to 250,181 and the Effective Bases range from 2,365,417 to 27,047,115. The Q20 and Q30 values are consistently above 95%, with GC content between 45.63 and 48.87%. These metrics suggest high sequencing accuracy and quality.

**TABLE 4:** Data preprocessing statistics and quality control analysis.

Sample name	Raw tags	Clean tags	Effective tags	Effective ratio (%)	Effective bases (nt)	Mean length	Q20 (%)	Q30 (%)	GC (%)
A1	103,612	71,862	59,818	57.73	14,089,175	236	99.87	99.46	48.87
A2	104,249	75,888	61,009	58.52	14,377,226	236	99.89	99.47	48.87
A3	84,640	64,818	50,566	59.74	11,924,510	236	99.88	99.44	48.8
B1	73,086	18,139	10,281	14.07	2,365,417	230	99.7	98.94	47.43
B2	208,456	69,919	50,471	24.21	12,107,437	240	98.86	97.01	47.83
B3	229,559	68,766	52,296	22.78	12,401,483	237	98.87	96.98	47.26
C1	242,973	111,736	88,742	36.52	21,109,742	238	99.48	98.22	48.3
C2	250,181	96,786	87,191	34.85	20,284,926	233	99.58	98.49	48.67
C3	116,391	39,474	32,681	28.08	7,964,340	244	99.1	97.44	47.26
D1	69,575	61,242	35,734	51.36	8,221,897	230	99.69	98.98	46.13
D2	217,648	189,190	121,181	55.68	27,047,115	223	99.61	98.47	45.63
D3	62,018	52,742	30,715	49.53	7,190,020	234	99.6	98.74	46.36

Table 5 reveals that the observed ASVs in these samples range from 31 to 131, with Shannon indices between 0.951 and 2.887 and Simpson indices ranging from 0.393 to 0.864. This analysis reveals that the fungal community in the sample exhibits high species richness and evenness, although its diversity is lower compared to bacteria. The Good’s coverage index of 1 suggests that the sequencing depth is sufficient to accurately represent the entire fungal community.

**TABLE 5:** Alpha diversity statistics table based on ASV.

Sample	Observed ASV	Shannon	Simpson	Chao1	ACE	Goods coverage
A1	41	0.953	0.393	43	43.719	1
A2	35	0.951	0.431	36.667	37.574	1
A3	31	0.992	0.444	31.333	31.776	1
B1	52	2.327	0.829	52	52	1
B2	131	2.887	0.864	132	131.299	1
B3	117	2.751	0.858	117	117.198	1
C1	61	1.31	0.503	63	63.442	1
C2	46	1.131	0.49	47.5	46.884	1
C3	45	1.91	0.688	45	45	1
D1	68	2.205	0.831	74	69.068	1
D2	64	2.165	0.82	66.5	66.806	1
D3	61	2.012	0.792	61	61.223	1

#### 3.3.2 Composition and differences in fungal communities

**FIGURE 3:**
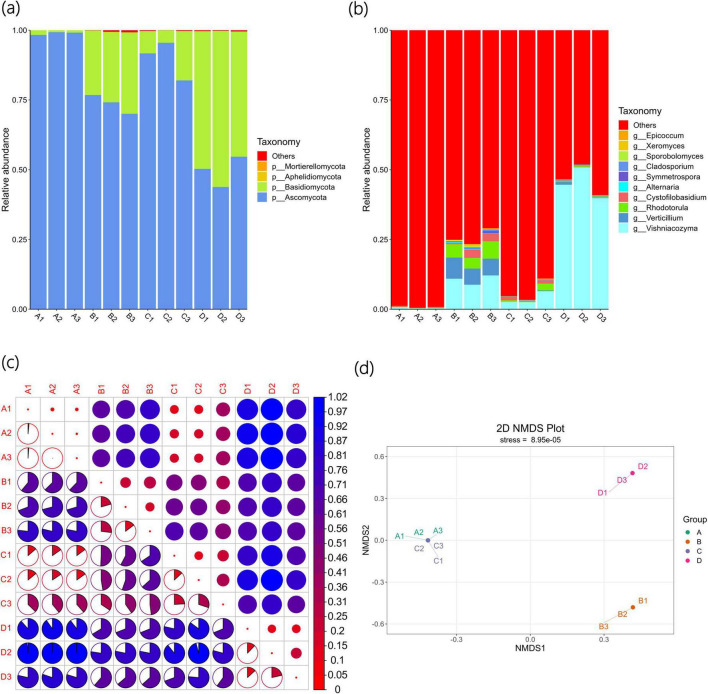
Beta diversity indices of fungal community of Agricultural Jiaosu samples after adding different bacterial agents at the phylum level **(a)**, at the genus level **(b)**, and based on ASV **(c)** heatmaps of Beta diversity indices, and **(d)** non-metric multi-dimensional scaling analysis.

As shown in Figure 3a, the four fungal phyla with the largest number of Agricultural Jiaosu sample types are *Ascomycota*, *Basidiomycota*, *Aphelidiomycota*, and *Mortierellomycota*. Among the four samples, the main fungal phyla are *Ascomycota* and *Basidiomycota*, with higher abundances in Group A and Group C. The abundances of *Basidiomycota* in Group B and Group D are higher than those in Group A and Group C, especially in Group D, where the abundance of *Basidiomycota* is the highest. This indicates that *Ascomycota* and *Basidiomycota* are the dominant phyla in Agricultural Jiaosu, and the proportion of fungal phyla will change depending on the different inoculants added before fermentation.

As shown in Figure 3b, the top 10 richest fungal genera are *Vishniacozyma*, *Verticillium*, *Rhodotorula*, *Cystofilobasidium*, *Alternaria*, *Symmetrospora*, *Cladosporium*, *Sporobolomyces*, *Xeromyces*, and *Epicoccum*. Overall, the abundances of *Vishniacozyma*, *Verticillium*, and *Rhodotorula* are the highest, suggesting that they may be the dominant genera in Agricultural Jiaosu. It is worth noting that, due to the complexity of the samples and the database, the majority of the relative abundance results show that the dominant fungal genera are others. The fungal genera of *Vishniacozyma* in Group B and Group D account for a relatively higher proportion. This indicates that the overall fungal species in Agricultural Jiaosu fermented with Chinese cabbage are similar, but the dominant fungal genera will vary after adding different inoculants before fermentation, especially in Groups AC and BD, where there are significant differences. The fungi of the *Vishniacozyma* genus have the ability to antagonize various plant pathogens and can be used for biological control to reduce fruit diseases. At the same time, they may enhance the plant’s disease resistance through endophytic characteristics. However, the fungi of the *Verticillium* genus mostly belong to plant pathogens, and there are a small number of *Verticillium* genera in Group B. It is uncertain whether they are harmful to crops in production. Therefore, when fermenting Agricultural Jiaosu, attention should be paid to factors such as fermentation environment and the types of inoculants added to prevent the occurrence of pathogenic bacteria.

The Beta diversity index heatmap based on ASV is shown in Figure 3c. This figure has the same meaning as Figure 2c. It can be seen that there are differences in fungal composition among each group, but compared with other groups, the differences between Group A and Group C are relatively small. This indicates that the fungal composition of Group A and Group C is the most similar among all groups. These results are also similar to the Non-Metric Multi-Dimensional Scaling (NMDS) analysis shown in Figure 3d. In this analysis, Group A and Group C are closer to each other and farther from the other two groups, further proving that except for the fungal composition of Group A and Group C being similar, the fungal composition differences among other groups are significant.

### 3.4 Analysis of organic acids in Agricultural Jiaosu

#### 3.4.1 Sample quality control analysis and PCA analysis

**FIGURE 4:**
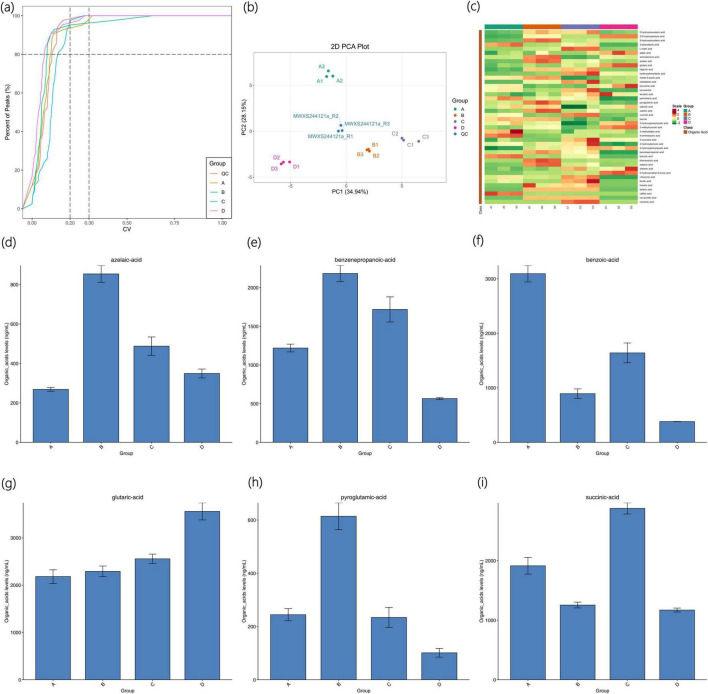
Distribution map of CV in each sample group of Agricultural Jiaosu after adding different microbial agents **(a)**, principal component analysis (PCA) map **(b)**, overall cluster analysis map **(c)**, column chart of *azelaic acid* content **(d)**, column chart of *benzenepropanoic acid* content **(e)**, column chart of *benzoic acid* content **(f)**, column chart of *glutaric acid* content **(G)**, column chart of *pyroglutamic acid* content **(h)**, and column chart of *succinic acid* content **(i)**.

The CV value, also known as the Coefficient of Variation, is the ratio of the standard deviation of the original data to the average of the original data, which can reflect the degree of data dispersion. The empirical cumulative distribution function (ECDF) can be used to analyze the frequency of substances with CV values less than the reference value. The proportion of substances with lower CV values in QC samples is higher, indicating that the experimental data is more stable: the proportion of substances with QC sample CV values less than 0.3 is higher than 80%, suggesting that the experimental data is stable; the proportion of substances with QC sample CV values less than 0.2 is higher than 80%, indicating that the experimental data is very stable. As shown in Figure 4a, the proportion of substances with CV values less than 0.2 in each sample is all higher than 80%, indicating that the experimental data is very stable.

By conducting principal component analysis on the samples (including QC samples), an initial understanding of the overall metabolic differences among each group of samples and the degree of variation among samples within each group can be obtained. As shown in Figure 4b, the PCA results show that the separation trend of the metabolic profiles among each group is good, and there are significant differences among each group.

#### 3.4.2 Analysis of organic acid types and contents

Four groups of Agricultural Jiaosu samples were subjected to cluster analysis. The horizontal axis represents the sample names and the vertical axis represents the metabolite information. Different colors represent different values obtained after normalization and filling (red indicates high content and green indicates low content). The results are shown in Figure 4c. A total of 42 organic acids were detected in the samples, among which the organic acids with higher content or significant differences between groups mainly include *glutaric acid*, *benzoic acid*, *succinic acid*, benzenepropanoic *acid*, azelaic *acid*, *pyroglutamic acid*, etc. The contents of these organic acids in each group were made into column charts, and the resulting figures are shown in Figures 4d–i.

As shown in Figure 4d, the content of azelaic *acid* was the highest in Group B and the lowest in Group A. Azelaic *acid* is less commonly applied in agriculture, but it can form stable complexes with metal ions in the soil, helping to dissolve minerals in the soil and thereby enhancing the bioavailability of certain nutrient elements. As shown in Figure 4e, the content of benzenepropanoic *acid* was the highest in Group B and the lowest in Group D. Benzenepropanoic *acid* can be one of the components of soil amendment agents and can improve soil structure by regulating soil pH and nutrient supply. As shown in Figure 4f, the content of *benzoic acid* was the highest in Group A and the lowest in Group D. *Benzoic acid* can serve as a carbon source for soil microorganisms and participate in the decomposition and transformation of soil organic matter. As shown in Figure 4g, *glutaric acid* was the most abundant organic acid in the samples, with the highest content in Group A and the lowest in Group D. *Glutaric acid* may be a metabolic product of certain microorganisms, although it is less commonly applied in agriculture, it has the potential to form complexes with metal ions due to its molecular structure containing two carboxyl groups. Therefore, it may have the effect of promoting the dissolution of metal ions in the soil and facilitating the absorption of these nutrient elements by plants. As shown in Figure 4h, the content of *pyroglutamic acid* was the highest in Group B and the lowest in Group D. *Pyroglutamic acid* can serve as a nitrogen source for soil microorganisms and participate in the decomposition and transformation of soil organic matter. As shown in Figure 4i, *succinic acid* was the most abundant in Group C and the lowest in Group D. *Succinic acid* is an important intermediate in the tricarboxylic acid cycle in organisms and is less applied in agriculture, but it can serve as an intermediate in energy metabolism and participate in the growth and metabolic processes of microorganisms. The above analysis reveals that the organic acids with higher contents in the Agricultural Jiaosu samples derived from the fermentation of Chinese cabbage are less directly applied in agricultural production. Therefore, the fertilizer effect of Agricultural Jiaosu in agriculture is likely to be achieved indirectly through influencing the soil environment and other means.

### 3.5 The influence of applying Agricultural Jiaosu on the growth and yield of Chinese cabbage

Four groups of Agricultural Jiaosu samples were applied as substitutes for chemical fertilizers in the process of growing Chinese cabbage three times. The treatment without fertilizer application was set as the control group, and the treatment with compound fertilizer application was set as the compound fertilizer control group. The results are shown in Table 6. In the process of Chinese cabbage production, Application of Agricultural Jiaosu significantly promoted the growth of the plant height of Chinese cabbage, among which Group C had the best effect; each group of Agricultural Jiaosu application could significantly promote the growth of the leaf length of Chinese cabbage, among which Group C and Group B had the best effects; there was no significant promoting effect of each group of Agricultural Jiaosu application on the growth of the leaf width of Chinese cabbage; each group of Agricultural Jiaosu application could significantly promote the growth of the stem thickness of Chinese cabbage; only Group B of the Agricultural Jiaosu application had a significant promoting effect on the leaf number growth of Chinese cabbage; each group of Agricultural Jiaosu application could significantly promote the increase of Chinese cabbage yield.

**TABLE 6:** The influence of applying Agricultural Jiaosu on the growth and yield of Chinese cabbage.

Sample	Plant height/cm	Leaf length/cm	Leaf width/cm	Stem thickness/cm	Number of leaves	Fresh weight/kg
A	31.933 ± 2.542b	34.033 ± 0.153cd	18 ± 1.97a	6.6 ± 0.7a	8.333 ± 0.577b	0.533 ± 0.104a
B	33.3 ± 0.693b	41 ± 0.656ab	20.967 ± 1.242a	6.333 ± 0.153a	10.333 ± 0.577a	0.603 ± 0.021a
C	39.067 ± 1.242a	42.333 ± 4.735a	20.067 ± 2.255a	6.967 ± 0.907a	8.667 ± 0.577b	0.707 ± 0.19a
D	31.8 ± 2.443b	35.033 ± 2.409c	19.6 ± 1.308a	6.067 ± 0.416a	8.333 ± 0.577b	0.54 ± 0.056a
E	32.067 ± 1.504b	36.9 ± 2.042bc	21.733 ± 2.515a	6.367 ± 0.153a	8 ± 0b	0.66 ± 0.04a
CK	23.2 ± 4.551c	30.167 ± 2.601d	16.7 ± 4.59a	4.9 ± 0.2b	7.667 ± 0.577b	0.3 ± 0.026b

The data in the same column are marked with different lowercase letters to indicate significant differences at the 0.05 level.

## 4 Discussion

### 4.1 The relationship between microbial diversity and raw materials in Agricultural Jiaosu

Previous studies have shown that in Agricultural Jiaosu, the phyla with the highest abundance of bacteria ranked from high to low are *Firmicutes*, *Pseudomonadota*, Bacteroidetes, and *Actinobacteria*, etc. In the fungal community, the phyla with the highest abundance of fungi ranked from high to low are the *Ascomycota* phylum and the *Basidiomycota* phylum. *Lactobacillus* and *Aspergillus* are the most abundant bacterial and fungal genera, respectively (Gao et al., 2022). The fungal diversity at the phylum level is consistent with the results of this study, but the bacterial diversity at the phylum level is slightly different from the results of this study. At the phylum level, the abundance of bacteria in this study is from high to low *Pseudomonadota*, *Firmicutes*, *Campylobacterota*, *Bacteroidota*, and *Actinobacteria*. At the genus level, the differences are greater. The most abundant genera of bacteria and fungi at the genus level in this study are *Arcobacter*, and *Vishniacozyma*, respectively. These differences may be due to the different fermentation raw materials. In this study, the raw material used was the waste of Chinese cabbage, while in Gao’s study, the waste of medicinal plants was used as the raw material. Thus, it can be speculated that different fermentation raw materials lead to different dominant microbial species, and this difference is greater than the difference caused by adding different microbial strains. The results of this study show that the microbial diversity at the phylum level of Agricultural Jiaosu fermented with four different microbial strains is not significantly different, but the diversity at the genus level is significant. Group A has relatively more *Bacteroides*, while Group B and Group D have relatively more *Arcobacter* and *Vishniacozyma*, and Group C has more *Lysinibacillus*.

### 4.2 Relationship between microbial diversity and nutrient elements and fertilizer efficiency in Agricultural Jiaosu

The results of this study show that Group A has relatively more *Bacteroides*, while previous studies have shown that *Bacteroides* has excellent ability to degrade plant polysaccharides (Qu et al., 2025). Agricultural waste already contains many polysaccharide components. It can be speculated that the degradation effect of Group A should be the best. The results of the nutrient element analysis of Agricultural Jiaosu in this study can confirm this speculation. The content of total carbon and total nitrogen in Group A is the highest among all groups. Although the specific mechanism still needs further research, it can be preliminarily determined that if the decomposition of agricultural waste in the process of making Agricultural Jiaosu is to be more thorough, it can try to add lactic acid bacteria before fermentation.

In addition, the abundance of *Arcobacter* in the *Arcobacter* genus and the fungi in the *Vishniacozyma* genus in Group B and Group D are relatively high. *Arcobacter* usually exists in various water bodies and animals, but it can now be isolated from various environmental sources. In the agricultural environment, Brucella helps to decompose organic residues and maintain soil fertility (Wesley, 2014). The fungi in the *Vishniacozyma* genus have the ability to antagonize multiple plant pathogens and can be used for biological control (Nian et al., 2023). Further research is needed on the specific mechanism. However, if Agricultural Jiaosu is to be used for soil improvement or biological control in applications, it is advisable to consider adding *Acetobacter* or *Bacillus subtilis*. In the experiment on the fertilizer efficiency of Chinese cabbage, the effects of Group B and Group D in promoting the growth of Chinese cabbage were generally not as good as those of the other groups. This might be due to the fact that soil improvement requires a long-term process. Therefore, in this short-term experiment, the promoting growth ability of Group B and Group D was not as good as that of the other groups.

In Group C of this study, *Lysinibacillus* was detected in large quantities. *Lysinibacillus* has great potential for application in agriculture, especially its role as a plant growth-promoting rhizobacteria (PGPR). The study found that bacteria in the *Lysinibacillus* genus can produce indole-3-acetic acid (IAA) and promote plant growth through nitrogen fixation, secretion of various enzymes to decompose organic matter, and release nutrients (Pantoja-Guerra et al., 2023). This is also consistent with the results of the fertilizer efficiency experiment of Chinese cabbage. In Group C of the experiment, Agricultural Jiaosu was the most effective in promoting the growth of Chinese cabbage in terms of plant height and leaf length. Although the specific mechanism still needs further exploration, if the purpose of using Agricultural Jiaosu is to promote vegetable growth as a fertilizer, it is advisable to add a little *Yeast* before fermentation. Since Agricultural Jiaosu is a mixture, it is not excluded that there is randomness in the fermentation process. Therefore, the specific mechanism still needs to be further studied under controlled conditions to determine.

The detection of Verticillium (a plant pathogenic genus) in Group B (relative abundance < 2%) underscores the importance of biosecurity measures during AJ production. To mitigate pathogen risks, strict control of fermentation conditions and pre-sterilization of raw materials (Lu et al., 2022) should be implemented. Additionally, inoculant screening for antimicrobial activity against plant pathogens, as demonstrated by Gao et al. (2022) could further enhance the safety of AJ for agricultural use.

### 4.3 Relationship between microbial diversity and organic acid metabolites in Agricultural Jiaosu

In this study, a total of 42 organic acids were detected in Agricultural Jiaosu. The top 6 organic acids in terms of content from high to low were *glutaric acid*, *benzoic acid*, *succinic acid*, benzenepropanoic *acid*, azelaic *acid*, and *pyroglutamic acid*. When comparing among the 4 groups, the content of *benzoic acid* in Group A was higher than that in other groups. In terms of microbial abundance, Group A had more *Bacteroides*. In this study, there was a positive correlation between *benzoic acid* and *Bacteroides*. Previous studies have shown that *benzoic acid* has antibacterial properties and can reduce the diversity of the cecal microbiota in the animal intestine (Diether et al., 2022). In this study, *benzoic acid* may inhibit the growth of other bacteria by promoting the increase of *Bacteroides*. It is also possible that *Bacteroides* evolved and metabolized to produce more *benzoic acid*, which in turn inhibited the growth of other bacteria. While the current study identified correlations between Bacteroides dominance and elevated benzoic acid levels in Group A, the causative metabolic pathways require further validation. Future investigations involving controlled co-culture systems could clarify whether Bacteroides directly produce benzoic acid or modulate its accumulation through substrate competition (Diether et al., 2022). Additionally, molecular characterization (e.g., metatranscriptomics) is needed to link microbial activity to organic acid profiles.

In Group B, the contents of benzenepropanoic *acid*, azelaic *acid*, and *pyroglutamic acid* were higher than those in other groups. They are all organic acids containing carboxyl groups. We speculate that there might be a metabolic complementarity relationship among them. The acetic acid produced by the acetic acid bacteria added before fermentation can be utilized by *Arcobacter* or *Vishniacozyma* or other microorganisms, and the organic acids produced by other microorganisms can also be utilized by the acetic acid bacteria. Then, the metabolic products (such as organic acids) of the microorganisms can serve as nutrients for other microorganisms, thereby promoting their growth. For instance, the accumulation of organic acids such as benzenepropanoic *acid*, azelaic *acid*, and *pyroglutamic acid* can provide carbon sources and energy for *Arcobacter* or *Vishniacozyma*. The content of *succinic acid* in Group C is higher than that in other groups, which is relatively easy to understand because *succinic acid* is an organic acid produced by *Yeast* at the early stage of alcohol fermentation and is an intermediate in important metabolic pathways such as the tricarboxylic acid cycle (TCA), the succinate bypass and the GABA bypass (Torres-Guardado et al., 2024). Since Group C added *Yeast* before fermentation, the content of *succinic acid* in Group C is higher than that in other groups. The content of *glutaric acid* in Group D is higher than that in other groups. *Glutaric acid* is a widely used chemical, and it is used in the consumer goods, textile, and footwear industries. The production methods of *glutaric acid* include various petroleum-based chemical methods, such as nitric acid catalyzed 2-cyano-cyclopentanone oxidation and acrylonitrile with ethyl succinate condensation (Han et al., 2020). There are more *Arcobacter* and *Vishniacozyma* in Group D, but no research has directly indicated the direct relationship between these microorganisms and *glutaric acid*. Moreover, because the content of *glutaric acid* in the other three groups is not low either, we speculate that the high content of *glutaric acid* in Group D is likely caused by the combined action of multiple microbial communities.

### 4.4 Relationship between organic acid metabolites in Agricultural Jiaosu and nutrients and fertilizer efficiency

In the results of this study, several organic acids in Agricultural Jiaosu, such as *glutaric acid*, *benzoic acid*, *succinic acid*, benzenepropanoic *acid*, azelaic *acid*, and *pyroglutamic acid*, are mostly applied in the chemical and pharmaceutical fields and rarely in the agricultural field. However, many organic acids have bactericidal and antibacterial effects (Diether et al., 2022; Chen et al., 2023). We speculate that the organic acids in Agricultural Jiaosu can be more applied in agricultural biological control rather than just as fertilizers. At the same time, we speculate that because some organic acids can serve as carbon sources that plants can absorb and utilize, in Group A with a high total carbon content, the content of *benzoic acid* is also relatively high, so *benzoic acid* may be more likely to be used as a carbon source in agricultural production. Furthermore, in the group C where the fertilizer effect was relatively good, the content of *succinic acid* was also relatively high. Therefore, we speculate that *succinic acid* is more likely to function as an immediate and short-term fertilizer effect agent for plant growth regulation rather than as a soil conditioner for long-term effects. Organic acids are the main substances in Agricultural Jiaosu, with complex structures, numerous types, and powerful effects. To fully understand their functions, further in-depth research is needed.

## 5 Conclusion

This study utilized the waste of Chinese cabbage as the raw material and investigated the effects of adding different microbial agents before fermentation on the nutrient elements, microbial diversity, organic acid content, and fertilizer efficiency of Agricultural Jiaosu. The results indicated that there were significant differences in the above aspects among the treatments with different microbial agents. Among them, the treatment group with *Lactobacillus* addition had the highest total carbon, total nitrogen, and total phosphorus contents; the treatment group with *Acetobacter* addition had the highest total potassium and carbon-nitrogen ratio. The microbial diversity analysis revealed that the *Bacteroides* relative abundance was higher in the *Lactobacillus* treatment group; *Arcobacter* and *Vishniacozyma* were relatively more abundant in the *Acetobacter* and *Bacillus subtilis* treatment groups; and the *Lysinibacillus* relative abundance was higher in the *Yeast* treatment group. In terms of organic acid content, the *benzoic acid* content was higher in the *Lactobacillus* treatment group; the benzenepropanoic *acid*, azelaic *acid*, and *pyroglutamic acid* contents were higher in the *Acetobacter* treatment group; the *succinic acid* content was the highest in the *Yeast* treatment group; and the *glutaric acid* content was the highest in the *Bacillus subtilis* treatment group. Moreover, Although the highest fresh weight increase with Agricultural Jiaosu (0.707 kg in Group C) was comparable to chemical fertilizer (0.660 kg), Agricultural Jiaosu demonstrates potential as a sustainable alternative by diverting vegetable waste from landfills and reducing synthetic fertilizer dependency.

In conclusion, Agricultural Jiaosu is rich in abundant nutrient elements, diverse microbial communities, and various organic acids, and has a significant fertilizer efficiency effect on Chinese cabbage production. Adding different microbial agents before fermentation would lead to differences in the composition and function of the finished Agricultural Jiaosu products. Therefore, appropriate microbial agents can be selected for fermentation according to specific application goals to optimize the performance and application effect of Agricultural Jiaosu.

## Data Availability

The datasets presented in this study can be found in online repositories. The names of the repository/repositories and accession number(s) can be found in the article/supplementary material.

## Footnotes

1
https://sciex.com.cn/

2
https://sciex.com/

## References

[B1] Abeydeera L. H. U. W. Mesthrige J. W. Samarasinghalage T. I. (2019). Global research on carbon emissions: A scientometric review. *Sustainability* 11:3972. 10.3390/su11143972

[B2] ASTM International (2016). *Standard Test Method for Total Nitrogen, and Total Kjeldahl Nitrogen (TKN) by Calculation, in Water by High Temperature catalytic Combustion and Chemiluminescence Detection.* West Conshohocken, PA: ASTM International.

[B3] Bengtsson-Palme J. Ryberg M. Hartmann M. Branco S. Wang Z. Godhe A. (2013). Improved software detection and extraction of ITS1 and ITS2 from ribosomal ITS sequences of fungi and other eukaryotes for analysis of environmental sequencing data. *Methods Ecol. Evol.* 4 914–919. 10.1111/2041-210X.12073

[B4] Caporaso J. G. Lauber C. L. Walters W. A. Berg-Lyons D. Huntley J. Fierer N. (2012). Ultra-high-throughput microbial community analysis on the Illumina HiSeq and MiSeq platforms. *ISME J.* 6 1621–1624. 10.1038/ismej.2012.8 22402401 PMC3400413

[B5] Caporaso J. G. Lauber C. L. Walters W. A. Berg-Lyons D. Lozupone C. Turnbaugh P. (2011). Global patterns of 16S rRNA diversity at a depth of millions of sequences per sample. *Proc. Natl. Acad. Sci.* 108 4516–4522. 10.1073/pnas.1000080107 20534432 PMC3063599

[B6] Chekri R. Noël L. Vastel C. Millour S. Kadar A. Guérin T. (2010). Determination of calcium, magnesium, sodium, and potassium in foodstuffs by using a microsampling flame atomic absorption spectrometric method after closed-vessel microwave digestion: Method validation. *J. AOAC Int.* 93 1888–1896. 10.1093/jaoac/93.6.1888 21313817

[B7] Chen H. Yu F. Kang J. Li Q. Warusawitharana H. Li B. (2023). Quality chemistry, physiological functions, and health benefits of organic acids from tea (Camellia sinensis). *Molecules* 28:2339. 10.3390/molecules28052339 36903584 PMC10005573

[B8] Cook A. M. Daughton C. D. (1981). Total phosphorus determination by spectrophotometry. *Methods Enzymol.* 72 292–295. 10.1016/S0076-6879(81)72017-2 7311835

[B9] Degnan P. H. Ochman H. (2012). Illumina-based analysis of microbial community diversity. *ISME J.* 6 183–194. 10.1038/ismej.2011.74 21677692 PMC3246231

[B10] Diether N. E. Nam S. L. Fouhse J. Le Thanh B. V. Stothard P. Zijlstra R. T. (2022). Dietary *benzoic acid* and supplemental enzymes alter fiber-fermenting taxa and metabolites in the cecum of weaned pigs. *J. Anim. Sci.* 100:skac324. 10.1093/jas/skac324 36205053 PMC9683507

[B11] Doyle J. J. Doyle J. L. (1987). A rapid DNA isolation procedure for small quantities of fresh leaf tissue. *Phytochem. Bull.* 19 11–15.

[B12] Duan Y. Wang Z. Ganeshan P. Sar T. Xu S. Rajendran K. (2025). Anaerobic digestion in global bio-energy production for sustainable bioeconomy: Potential and research challenges. *Renewable Sustainable Energy Rev.* 208:114985. 10.1016/j.rser.2024.114985

[B13] Findley K. Oh J. Yang J. Conlan S. Deming C. Meyer J. (2013). Topographic diversity of fungal and bacterial communities in human skin. *Nature* 498 367–370. 10.1038/nature12171 23698366 PMC3711185

[B14] Gao Y. Zhang Y. Cheng X. Zheng Z. Wu X. Dong X. (2022). Agricultural Jiaosu: An eco-friendly and cost-effective control strategy for suppressing Fusarium root rot disease in Astragalus membranaceus. *Front. Microbiol.* 13:823704. 10.3389/fmicb.2022.823704 35432283 PMC9008360

[B15] Gao Y. Zheng Z. Cheng X. Zhang Y. Liu X. Hu Y. (2023). An innovative way to treat cash crop wastes: The fermentation characteristics and functional microbial community using different substrates to produce Agricultural Jiaosu. *Environ. Res.* 227 :115727. 10.1016/j.envres.2023.115727 36948282

[B16] Grizzetti B. Bouraoui F. Billen G. Cardoso A. C. Thoss V. Van Drecht G. (2022). Agricultural nitrogen and phosphorus pollution: A global assessment of risks and mitigation options. *Nat. Sustainabil.* 5 704–714. 10.1038/s41893-022-00847-x

[B17] Han T. Kim G. B. Lee S. Y. (2020). *glutaric acid* production by systems metabolic engineering of an l-lysine-overproducing Corynebacterium glutamicum. *Proc. Natl. Acad. Sci.* 117 30328–30334. 10.1073/pnas.2017483117 33199604 PMC7720191

[B18] Hess M. Sczyrba A. Egan R. Kim T. W. Chokhawala H. Schroth G. (2011). Metagenomic discovery of biomass-degrading genes and genomes from cow rumen. *Science* 331 463–467. 10.1126/science.1200387 21273488

[B19] Lu X. Yang Y. Hong C. Zhu W. Yao Y. Zhu F. (2022). Optimization of vegetable waste composting and the exploration of microbial mechanisms related to fungal communities during composting. *J. Environ. Manag.* 319:115694. 10.1016/j.jenvman.2022.115694 35841778

[B20] National Bureau of Statistics of China. (2023). *National*] *Statistics Data of China.* Available online at: https://data.stats.gov.cn/easyquery.htm?cn=C01 (accessed February, 2025).

[B21] Nian L. Xie Y. Zhang H. Wang M. Yuan B. Cheng S. (2023). *Vishniacozyma* victoriae: An endophytic antagonist *Yeast* of kiwifruit with biocontrol effect to Botrytis cinerea. *Food Chem.* 411:135442. 10.1016/j.foodchem.2023.135442 36652885

[B22] Oros-Sichler M. Smalla K. (2013). “Semi-nested PCR approach to amplify large 18S rRNA gene fragments for PCR-DGGE analysis of soil fungal communities,” in *Laboratory Protocols in Fungal Biology: Current Methods in Fungal Biology*, eds Gupta V. K. Tuohy M. G. Saxena R. C. Bennett J. W. Batt C. A. Tortorello M. L. (Berlin: Springer), 289–298. 10.1007/978-1-4614-2356-0_23

[B23] Pace N. R. (1997). A molecular view of microbial diversity and the biosphere. *Science* 276 734–740. 10.1126/science.276.5313.734 9115194

[B24] Pantoja-Guerra M. Burkett-Cadena M. Cadena J. Dunlap C. A. Ramírez C. A. (2023). *Lysinibacillus* spp.: An IAA-producing endospore forming-bacteria that promotes plant growth. *Antonie van Leeuwenhoek* 116 615–630. 10.1007/s10482-023-01828-x 37138159 PMC10257616

[B25] Puglia D. Pezzolla D. Gigliotti G. Torre L. Bartucca M. L. Del Buono D. (2021). The opportunity of valorizing agricultural waste, through its conversion into biostimulants, biofertilizers, and biopolymers. *Sustainability* 13:2710. 10.3390/su13052710

[B26] Qu Z. Liu H. Yang J. Zheng L. Huang J. Wang Z. (2025). Human gut *Bacteroides* and Para*Bacteroides* species selectively utilize medicinal polysaccharides. *Nat. Commun.* 16:55845. 10.1038/s41467-025-55845-7 39809740 PMC11733155

[B27] Shetty A. Goyal A. (2022). Total organic carbon analysis in water – A review of current methods. *Materials Today Proc.* 65 3881–3886. 10.1016/j.matpr.2022.07.173

[B28] Su Y. Liu C. Fang H. Zhang D. (2020). *Bacillus subtilis*: A universal cell factory for industry, agriculture, biomaterials and medicine. *Microb. Cell Factories* 19:173. 10.1186/s12934-020-01436-8 32883293 PMC7650271

[B29] Tang Y. Dong J. Gruda N. Jiang H. (2023). China requires a sustainable transition of vegetable supply from area-dependent to yield-dependent and decreased vegetable loss and waste. *Int. J. Environ. Res. Public Health* 20:1223. 10.3390/ijerph20021223 36673990 PMC9859069

[B30] Torres-Guardado R. Rozès N. Esteve-Zarzoso B. Reguant C. Bordons A. (2024). *succinic acid* production by wine *Yeast*s and the influence of GABA and glutamic acid. *Int. Microbiol.* 27 505–512. 10.1007/s10123-023-00410-9 37498437 PMC10990983

[B31] Wan W. Wang Y. Tan J. Qin Y. Zuo W. Wu H. (2020). Alkaline phosphatase-harboring bacterial community and multiple enzyme activity contribute to phosphorus transformation during vegetable waste and chicken manure composting. *Bioresour. Technol.* 297:122406. 10.1016/j.biortech.2020.122406 31787513

[B32] Wang R. Min J. Kronzucker H. J. Li Y. Shi W. (2019). N and P runoff losses in China’s vegetable production systems: Loss characteristics, impact, and management practices. *Sci. Total Environ.* 663 971–979. 10.1016/j.scitotenv.2019.01.368 30739865

[B33] Wesley I. V. (2014). “*Arcobacter*: An emerging pathogen and its role in the environment,” in *Encyclopedia of Food Microbiology*, 2nd Edn, eds Batt C. A. Tortorello M. L. (Amsterdam: Elsevier).

[B34] Xing T. Yu S. T. Zhen F. Kong X. Sun Y. (2020). Anaerobic fermentation of hybrid Pennisetum mixed with fruit and vegetable wastes to produce volatile fatty acids. *RSC Adv.* 10 33261–33267. 10.1039/D0RA03424A 35515045 PMC9056692

[B35] Ye P. Xiong J. Wu X. Liu H. Han L. Huang G. (2023). Insights into carbon loss reduction during aerobic composting of organic solid waste: A meta-analysis and comprehensive literature review. *Sci. Total Environ.* 10.1016/j.scitotenv.2023.163138 Online ahead of print. 36502991

[B36] Youssef N. Sheik C. S. Krumholz L. R. Najar F. Z. Roe B. A. Elshahed M. S. (2009). Comparison of species richness estimates obtained using nearly complete fragments and simulated pyrosequencing-generated fragments in 16S rRNA gene-based environmental surveys. *Appl. Environ. Microbiol.* 75 5227–5236. 10.1128/AEM.00592-09 19561178 PMC2725448

[B37] Zaini N. S. M. Khudair A. J. D. Mohsin A. Z. Lim E. J. Minato W. Idris H. (2023). Biotransformation of food waste into biofertilisers through composting and anaerobic digestion: A review. *Plant Soil Environ.* 69 409–420. 10.17221/101/2023-PSE

[B38] Zhang F. Li L. Cui Z. Chen X. Zhang W. (2021). Impacts of excessive fertilizer use on soil health and crop productivity: A review. *Agron. Sustainable Dev.* 41 1–16. 10.1007/s13593-020-00654-2

[B39] Zhang Y. Gao Y. Zheng Z. Meng X. Cai Y. Liu J. (2020). A microbial ecosystem: Agricultural Jiaosu achieves effective and lasting antifungal activity against Botrytis cinerea. *AMB Express* 10:216. 10.1186/s13568-020-01156-7 33315172 PMC7736446

